# Neural ageing and synaptic plasticity: prioritizing brain health in healthy longevity

**DOI:** 10.3389/fnagi.2024.1428244

**Published:** 2024-08-05

**Authors:** Sheeja Navakkode, Brian K. Kennedy

**Affiliations:** ^1^Healthy Longevity Translational Research Program, Yong Loo Lin School of Medicine, Centre for Healthy Longevity, National University Health System, National University of Singapore, Singapore, Singapore; ^2^Life Sciences Institute Neurobiology Programme, Centre for Life Sciences, National University of Singapore, Singapore, Singapore; ^3^Department of Physiology, Yong Loo Lin School of Medicine, National University of Singapore, Singapore, Singapore; ^4^Departments of Biochemistry, Yong Loo Lin School of Medicine, National University of Singapore, Singapore, Singapore; ^5^Buck Institute for Research on Ageing, Novato, CA, United States

**Keywords:** healthy ageing, cognitive ageing, longevity, synaptic plasticity, hippocampus, calcium, long-term potentiation, neuroinflammation

## Abstract

Ageing is characterized by a gradual decline in the efficiency of physiological functions and increased vulnerability to diseases. Ageing affects the entire body, including physical, mental, and social well-being, but its impact on the brain and cognition can have a particularly significant effect on an individual’s overall quality of life. Therefore, enhancing lifespan and physical health in longevity studies will be incomplete if cognitive ageing is over looked. Promoting successful cognitive ageing encompasses the objectives of mitigating cognitive decline, as well as simultaneously enhancing brain function and cognitive reserve. Studies in both humans and animal models indicate that cognitive decline related to normal ageing and age-associated brain disorders are more likely linked to changes in synaptic connections that form the basis of learning and memory. This activity-dependent synaptic plasticity reorganises the structure and function of neurons not only to adapt to new environments, but also to remain robust and stable over time. Therefore, understanding the neural mechanisms that are responsible for age-related cognitive decline becomes increasingly important. In this review, we explore the multifaceted aspects of healthy brain ageing with emphasis on synaptic plasticity, its adaptive mechanisms and the various factors affecting the decline in cognitive functions during ageing. We will also explore the dynamic brain and neuroplasticity, and the role of lifestyle in shaping neuronal plasticity.

## Highlights

- Synaptic Plasticity impairments contribute to the processes of neural ageing.
- Targeting adaptive mechanisms in neural plasticity can help to attenuate age-related cognitive decline.
- Altered hippocampal plasticity is a major driver to cognitive decline in ageing.
- Calcium dyshomoeostasis, neuroinflammation, autophagy and metabolic alterations are major contributors to ageing.
- Lifestyle factors influences neural plasticity and healthy ageing.

## Introduction

The brain, like any other biological system in the body, experiences physiological ageing, which is reflected by a decline in cognitive, social and motor abilities (Salthouse, 2012). It includes a decline in information processing, executive functions, planning and working memory (Park et al., 2002). Along with the decline in mental abilities, ageing also predisposes the brain to various diseases (Burch, 1978; Reeve et al., 2014; Niccoli et al., 2017; Hou et al., 2019). The term “benign forgetfulness of senescence” or “age-associated memory decline “was used to differentiate individuals undergoing memory decline due to ageing from those whose memory impairment is linked to neurological damage or disease (Kelley and Minagar, 2009).

Analysis of various model systems suggests that the genetic pathways regulating cognitive ageing is highly conserved in organisms ranging from yeast, worms, flies to mammals. More interestingly, these models indicate a dynamic association between cognitive functions and ageing (Barco et al., 2006; Ardiel and Rankin, 2010; Besson et al., 2010). Therefore, it is important to identify key regulators of both cognitive impairment and pathways related to longevity. Interventions directed at longevity pathways could potentially offer benefits in addressing cognitive decline and neurodegenerative conditions, and conversely, targeting cognitive health may also impact longevity pathways.

Unlike other systems in the body, a remarkable characteristic of the brain, is that it possess an intrinsic capacity to adapt to a rapidly changing environment which is called neuronal or synaptic plasticity (Benfenati, 2007). This activity-dependent plasticity is a unique feature of the nervous system, that allows it to change connections according to experiences (Martin et al., 2000). With ageing, the brain undergoes a gradual decline in its capacity to both physically and functionally adapt to changing or novel environments (Burke and Barnes, 2006). This gradual decline in plasticity is implicated in the functional decline of many cognitive functions and makes the brain vulnerable to neurodegenerative disorders like Alzheimer’s disease (AD), Parkinson’s disease (PD), and others, thus making it both the cause and target for interventions (Rowan et al., 2003; Bagetta et al., 2010; Mango et al., 2019). Therefore, in addition to alterations in brain structure and function during ageing, it seems that the mechanisms governing how structure and function can be altered also undergo changes throughout the lifespan.

Although significant advancements have been made in the understanding of mechanisms involved in synaptic plasticity, its influence on healthy brain ageing, lifespan extension and adaptive plasticity remains poorly understood. In this review, we will detail the mechanisms of cognitive decline, including brain’s adaptive mechanisms and dysfunction in synaptic plasticity that contribute to the ageing process, with an emphasis on the hippocampus and the factors that make it vulnerable to neurodegenerative disorders. We will also discuss the dynamic brain and the role of lifestyle changes that affect synaptic plasticity and a comparative account of ageing of human, rodents and non-human primates as models for ageing research.

### Adaptive mechanisms in ageing brain

Evidence from multiple sources, including neuroimaging, cognitive psychology, neuropathology, metabolic, cellular and molecular studies points to the adaptability and resilience of the ageing brain (Jack et al., 1997; Cabeza et al., 2018; Stern et al., 2019).These includes metabolic, physiological and neural network adaptations that improves cognitive, mental and physical wellbeing. Enhancing the adaptive mechanisms within neural cells could potentially protect or delay the ageing process. Moreover, a range of physiological and pharmacological interventions can activate these adaptive mechanisms, which include education, social interactions, a balanced diet, physical exercise, anti-inflammatory drugs, senolytics, antihypertensive drugs, reducing stress, and mental training like mindfulness (Aron et al., 2022). Collectively, it highlights that the ageing brain exhibits adaptive changes that could delay the onset of functional decline that accompanies brain ageing.

A very exciting study by Zullo et al. (2019) showed that a reduced neural activity and downregulation of genes that mediate excitatory neurotransmission is an adaptive mechanism that delays cognitive decline and/or increases lifespan. This is supported by the findings that targeting excess neural excitation may improve cognition and attenuate AD pathology in late-onset AD (Haberman et al., 2017; Zott et al., 2019). The restrictive element-1 silencing transcription factor (REST)/NRSF [neuron-restrictive silencing factor (NRSF)], a transcriptional repressor, represses excitation-related genes and is found to be upregulated in humans with extended longevity. Mice lacking REST show increased cortical activity and neuronal excitability with ageing. Similarly, mutations that reduce the function of the *Caenorhabditis elegans* (*C. elegans*) REST orthologs, spr-3 and spr-4, increase neural excitation and reduce the lifespan (Zullo et al., 2019). In *C. elegans*, neural excitation increases with age and inhibition of excitation, increases longevity. REST can be activated by a variety of stressors, including oxidative stress, DNA damage, and amyloid beta, reflecting the brain’s adaptive mechanisms (Lu et al., 2014). It also promotes neuroprotection by repressing genes involved in oxidative stress and β-amyloid toxicity (Hwang and Zukin, 2018). Thus, exploring potential targets to activate REST or drugs that reduce excitation (e.g., nemadipine and Ivermectin) could open up new avenues for the development of innovative therapeutic strategies to slow down ageing and reduce the risk for neurodegenerative disorders.

An increasing body of evidence suggests that metabolic adaptations within the body and brain have played significant roles in the evolution of the primate brain (Khaitovich et al., 2008; Pontzer et al., 2016; Camandola and Mattson, 2017). Loss of metabolic homeostasis is an important hallmark of ageing and is characterized by alteration of regulatory processes that controls cellular functions (López-Otín et al., 2013; Kennedy et al., 2014; Goh et al., 2023). The alteration of homeostasis with ageing is influenced by a complex interplay of factors, including damage to biological macromolecules induced by stress, changes in energy metabolism, deregulated cellular and tissue integrity, and a loss or decline of coordination of physiological control mechanisms (López-Otín et al., 2013). Simultaneously, there are conserved genetic mechanisms that have evolved to prevent or counteract this decline. Some of the highly conserved metabolic pathways that has adaptive roles during ageing, includes those mediated by insulin/insulin-like growth factor signaling (IIS), the mammalian target of rapamycin (mTOR), AMP-activated protein kinase (AMPK), and sirtuin enzymes (Burkewitz et al., 2014; Chang and Guarente, 2014; Papadopoli et al., 2019). These adaptive metabolic changes can be neuroprotective by decreasing inflammation, activating stress resistance factors, promoting autophagy or interfering with other molecular pathways that increase ageing processes. These adaptive mechanisms can be enhanced by changes in lifestyle like decreased stress, meditation, yoga, physical exercise, diet, non-invasive brain stimulations or drugs like rapamycin, alpha ketoglutarate, metformin, NAD+ precursors, and others (Mattson and Arumugam, 2018).

Metabolic dysregulation linked with obesity is similar to that observed in normal ageing, and several studies suggests that obesity could accelerate processes of ageing (Santos and Sinha, 2021). Obesity in ageing is associated with blood brain barrier disruption, oxidative stress, neuroinflammation, microglial activation and associated impairment of hippocampus-dependent learning and memory (Tucsek et al., 2014). An evolutionary perspective on why obesity or over consumption of food impairs cognition is that the neural pathways that have evolved to adapt to food scarcity are affected by the increased caloric intake and sedentary lifestyle, thus accelerating brain ageing (Tucsek et al., 2014). Nutritional regulation and drug therapy with anti-ageing gene modification can be a focus, where targeting anti-ageing genes like Sirtuin 1 (Sirt 1) Klotho, p66Shc (longevity protein) and Fork head box proteins (FOXO1/ FOXO3a) holds promise in the regulation of obesity, and associated cognitive decline to facilitate healthy ageing (Ciciliot and Fadini, 2019; Valcarcel-Ares et al., 2019; Behl et al., 2021; Orces, 2022). Implementing behavioral interventions like physical exercise, healthy diet, stress management, social engagement, sleep hygiene etc. offers the potential to mitigate the impact of obesity on brain health in ageing adults (Stillman et al., 2017). Physical exercise improves cognitive ageing by influencing cognition directly, or indirectly, by reducing obesity (Nelson et al., 2007). Interventions designed to optimize macro and micronutrients also can potentially form the basis for strategies aimed at improving cognitive functioning, particularly in the ageing brain (Melzer et al., 2021). Applying the six pillars of lifestyle medicine (LM), comprising plant-based nutrition, regular physical activity, stress management, avoidance of harmful substances, quality sleep, and fostering social connections, has the potential to enhance neurocognitive functions and supports ahealthy weight and overall well-being (Jaqua et al., 2023). Therefore, giving priority to lifestyle changes is crucial in promoting brain health, with a focus on preventing cognitive decline.

### Cognitive decline in normal ageing

Normal ageing is characterized by significant declines in cognitive performance especially on tasks necessitating rapid information processing, decision-making, speed of processing, working memory, and executive cognitive function (Murman, 2015). Decline or loss of brain functions, including learning and memory, and loss of ability to live independently is one of greatest fears of ageing. According to World Health Organisation (WHO), cognitive decline has become a major health concern in old age. These can only get larger as the average age of the population rises, therefore understanding the basis of cognitive decline during ageing is critical for healthy ageing.

Memory or cognition is not a unitary concept, but a complex, multifactorial process. Various memory components are influenced by the ageing process in different ways (Nilsson, 2003). Some mental functions, such as verbal ability, certain numerical skills and general knowledge experience minimal age-related decline (Deary et al., 2009). The language skills and vocabulary that are developed during young years are mostly retained. While a lot of mental abilities starts to decline from middle age onwards. Ageing primarily affects episodic memory rather than semantic memory, indicating that some facets of memory are affected more than others in normal ageing processes (Nilsson et al., 1997; Schaie and Willis, 2010). Episodic memory is the recollection of day-to-day events, such as ‘where we parked the car’ or ‘where we met our friend,’ while semantic memory is the retention of factual knowledge, such as ‘the capital of a country’. While decline in both semantic and episodic memories occurs in ageing, episodic memory progressively declines over age while semantic memory is affected only in late life (Rönnlund et al., 2005). Executive dysfunction is characterized by difficulties in planning, mental flexibility, inhibiting inappropriate actions, distinguishing relevant sensory information and problem solving (Stuss and Benson, 1984). Another important hallmark of ageing is the slowdown of information processing or decrease in the speed at which people perform decision making tasks (Salthouse and Ferrer-Caja, 2003). Attentive efficiency, which is the ability to sustain focussed attention and cognitive processing, is reduced with increasing age (Commodari and Guarnera, 2008). The ability to encode new information also declines with age, while retention of the already learned information is preserved in older adults (Carlson et al., 1995; Whiting and Smith, 1997). Visual construction skills, which represent the ability to assemble different parts to a whole like assembling the furniture also declines with age (Howieson et al., 1993). Age-related changes exhibit significant variability among individuals, with some people experiencing remarkable resilience, while others are more susceptible to decline and age-related diseases (Salthouse, 2012).

The decline in cognitive functions are not always accompanied by neuronal loss; instead, they are associated with synaptic changes like loss of synapses and connections or dysfunctions in synaptic plasticity (Morrison and Baxter, 2012). Animal models of ageing, including rodents and non-human primates, shows a functional decline in memory processes similar to humans (Small et al., 2004; Kelly et al., 2006; Crimmins, 2015). This decline in memory and plasticity is more evident in the hippocampus, which is more vulnerable to ageing and age-associated diseases (Bach et al., 1999; Wong et al., 2021).

### Ageing hippocampus and cognitive decline

One brain structure of significant relevance in ageing and cognitive decline is the hippocampus, a region known for its crucial involvement in the processes of learning and memory that is found deep within the medial temporal lobe of the brain (Bettio et al., 2017). Although functional magnetic resonance imageing (MRI) findings have shed light into the functional role of the hippocampus in various cognitive functions, the impact of subtle changes in hippocampal structure and function during normal healthy ageing remains poorly understood.

In the hippocampus, information processing occurs through the trisynaptic pathway, involving connections from the entorhinal cortex (EC) to the dentate gyrus (DG) via the perforant path. Subsequently, mossy fibers originating from the DG terminate in both the DG and CA3. From the CA3, fibers known as Schaffer collaterals project to CA1. Ultimately, CA1 sends outputs to the subiculum and the entorhinal cortex (EC) (Eichenbaum, 2004). Among the various subfields of the hippocampus, the CA1 is more susceptible to age-associated changes (Mueller et al., 2007).

Age related changes in the hippocampus include changes in gene expression patterns, altered intracellular signaling, increased oxidative stress, neuroinflammation, structural and functional changes in synapses, and impaired neurogenesis (Ojo et al., 2012; Berchtold et al., 2019; Hu and Li, 2020; Wang et al., 2023). A correlation has been observed in rats between age-related declines in hippocampal-dependent learning and memory and a reduction in hippocampal volume, highlighting the structural changes associated with cognitive decline in brain ageing (Driscoll et al., 2006). Hyperexcitability in hippocampal neurons is another key pathological change that renders the ageing brain vulnerable to neurodegenerative diseases (Targa Dias Anastacio et al., 2022).

Hippocampal transcriptional analysis shows distinctive gene expression changes associated with ageing (Stilling and Fischer, 2011). Expression of the DNA methyltransferase Dnmt3a2 declines in the ageing hippocampus, and genetic restoration of Dnmt3a2 expression mitigates the age associated cognitive decline (Oliveira et al., 2012). At the cellular level, hippocampal changes include alterations in glucose metabolism, oxidative stress, and autophagy. The neuronal glucose uptake is impaired in the ageing hippocampus, and the abundance of enzymes associated with glycolysis and gluconeogenesis declines with age (Freeman et al., 2009; Ding et al., 2013). Ageing is also associated with increased mitochondrial dysfunction leading to decreased capacity for ATP production and increased production of reactive oxygen species (ROS) (Weinreb et al., 2007; Navarro and Boveris, 2010).

The structural and functional alterations in the hippocampus are in line with the severity of age-related cognitive decline. Structural alterations in hippocampus with ageing include decreased neuronal count and number of synaptic connections (Issa et al., 1990; Geinisman et al., 1995; Raz et al., 2000). The subtle synaptic and functional changes within the hippocampus underlie the majority of age-related memory impairment (Morrison and Baxter, 2012). Thus, in general, age-related changes within the hippocampus contribute to a gradual loss of synaptic plasticity, which serves as a major driver of age-related functional and cognitive decline.

### Synaptic alterations in hippocampus

Donald Hebb, a Canadian neuropsychologist, proposed that increase in synaptic efficacy arise from a presynaptic cell’s repeated and persistent stimulation of a postsynaptic cell. His theory, called Hebb’s postulate, envisages that “the cells that fire together will wire together.” Accordingly, the coincident firing of pre and post synaptic neurons strengthens their connections. After two decades, Tim Bliss and Terje Lomo discovered a biological process that aligned with Hebb’s prediction, where a high frequency stimulation in the dentate gyrus of rabbits resulted in the strengthening of connections between two neurons resulting in long-term potentiation (LTP) (Bliss and Lomo, 1973; Lømo, 2003). LTD (long-lasting depression) is the persistent weakening of the synapses after a low frequency stimulation (Dudek and Bear, 1992). LTP and LTD of neural plasticity are the most widely studied cellular correlates of learning and memory (Malenka and Bear, 2004).

Alterations in hippocampal LTP are involved in age-related learning deficits. These age-related alterations in synaptic transmission have been studied in both animal models and human subjects (Anderson and Rutledge, 1996; Luebke and Chang, 2007; Dumitriu et al., 2010). Age-associated reductions in the induction and maintenance of LTP, along with an increased propensity for LTD, were observed in rodents. These findings provide a likely neural basis for memory decline associated with ageing (Deupree et al., 1993; Norris et al., 1996; Rosenzweig et al., 1997; Shetty et al., 2017; Wang et al., 2023).

**Figure 1:**
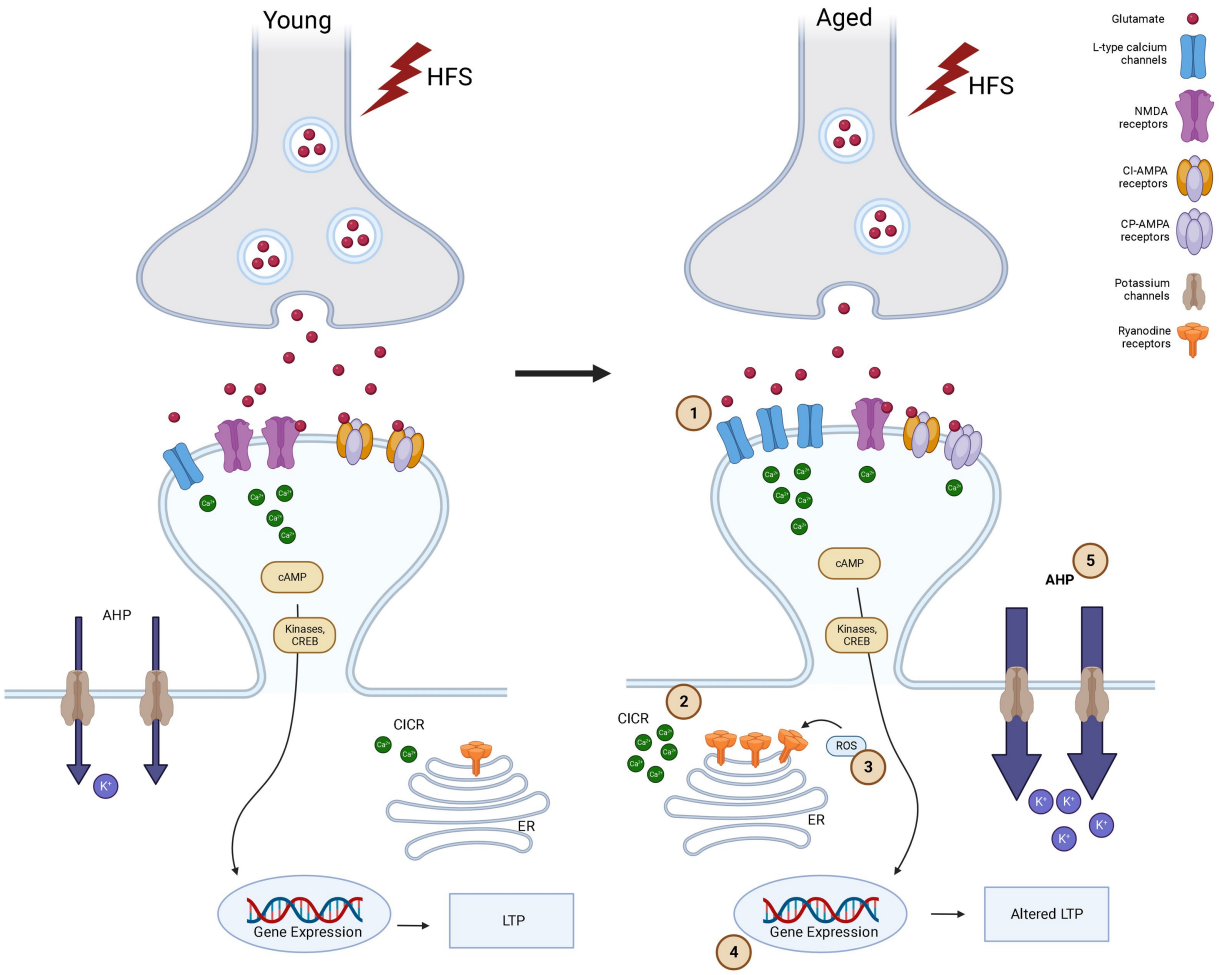
Synaptic plasticity alterations in young and aged synapses after high frequency stimulation (HFS).

Two forms of LTP coexist at Schaffer collateral (SC)-CA1 synapses, and both of these are subject to ageing. One form of LTP, driven by the activation of NMDA receptors (NMDA-receptor-LTP), tends to diminish in older rats (Rosenzweig and Barnes, 2003; Burke and Barnes, 2006). Conversely, the other type of LTP that depends on the activation of L-type voltage-gated calcium channels (VGCC-LTP) increases with advancing age (Shankar et al., 1998). Most interestingly, ageing reduces NMDA-receptor-dependent LTP across the spectrum of cognitive outcomes, whereas increased NMDA-receptor-independent LTP occurs exclusively in high-performing aged rats indicating that a shift to NMDA-receptor-independent pathways for neural plasticity is an adaptive mechanism with better cognitive outcomes in ageing (Boric et al., 2008). This shift in the mechanism of LTP is another characteristic of aged synapses (Figure 1). In rat hippocampal CA2 and CA3 regions, a significant decrease in NMDA receptor subunit GluN1 has been characterized in ageing (Liu et al., 2008).

Ageing alters NMDA-receptor-dependent mechanisms causing an increase in postsynaptic calcium levels, leading to altered LTP/LTD and memory functions (Thibault et al., 2001; Tombaugh et al., 2002; Burke and Barnes, 2006; Temido-Ferreira et al., 2020). Several studies have showed a decline in the late phase of NMDA-receptor-dependent LTP, in aged animals (Barnes, 2003; Huang and Kandel, 2006; Foster, 2012). An age associated decrease in reelin, a protein that interact with NMDA receptors can lead to cognitive decline and impaired LTP in ageing (Doehner and Knuesel, 2010). Reelin can regulate NMDA receptors through phosphorylation of GluN2 subunits, and modulate LTP (Doehner and Knuesel, 2010). Similar effects are observed with deletion of glycine transporter1 that enhances synaptic glycine and NMDA functions (Dubroqua et al., 2010). The neural cell adhesion molecule (NCAM), a neuronal surface glycoprotein involved brain plasticity associated with learning and memory, plays a role in NMDA receptor trafficking and the inhibition of extra synaptic NMDA receptors containing GluN2B subunits. During the ageing process, deficits in neural cell adhesion molecule function leads to impaired LTP and LTD. These deficits can potentially be reversed by d-cycloserine, primarily acting through NMDA receptors that contain GluN2A subunits (Kochlamazashvili et al., 2012). Proteomic studies reveal an age-dependent decrease in synaptophysin, postsynaptic density 95, and soluble NSF attachment protein receptor (SNARE) family protein expression (VanGuilder et al., 2010).

Structural changes in the hippocampus, such as synaptic loss, one of the hallmarks of normal ageing, also play an important role in age-associated cognitive decline. It has been shown that approximately a 20–40% reduction in dendritic spines in the cortex and hippocampus was observed during ageing in both humans and experimental animals. This loss of dendritic spines is consistent with a reduction in the field excitatory postsynaptic potential in aged rats (Barnes, 1979; Barnes and Mcnaughton, 1980; Luebke et al., 2004). Aged rats with spatial learning deficits showed a reduction in the size of post synaptic densities of perforated synapses in the CA1 area of hippocampus. This shows that impairment of hippocampus-dependent learning tasks is associated with structural alterations in synapses (Nicholson et al., 2004).

In aged monkeys, while the density of perforated synapses in the dentate gyrus is preserved in old relative to young monkeys, a reduction in synaptic contacts per axonal bouton was associated with cognitive impairment (Haberman et al., 2011). An age-related increase in the non-synaptic boutons in the molecular layer that receives input from EC in aged monkeys impacts cognition (Hara et al., 2011). Age-related decrease in NMDA and AMPA-receptors were also reported in the hippocampus. Gene expression profiling studies have showed a downregulation of genes associated with synaptic transmission and plasticity in hippocampal CA3 in aged rats (Haberman et al., 2011). Comparison of the spatial firing patterns of CA1 and CA3 neurons in aged memory impaired rats showed that aged CA3 cells failed to rapidly encode new spatial information compared with young CA3 cells while CA1 place cells in aged and young rats had similar firing characteristics (Wilson et al., 2005).

These studies shows that alterations in the synaptic plasticity characteristics of hippocampal neurons significantly contribute to the observed changes in neural dynamics during the ageing process. Therefore, gaining insight into the causal factors that contribute to the underlying cellular and molecular changes during ageing will be instrumental in the development of therapeutic interventions.

Cartoon showing the signaling pathways in synaptic plasticity in Young versus Aged synapses in CA1 area of hippocampus after high frequency stimulation. High frequency stimulation (represented as lightning bolts) leads to release of glutamate. In response to glutamate release, in flow of Ca^2+^ occurs in to the post synaptic cell via NMDA-receptors and LTCCs. This calcium increase in the post synaptic cell activates the phosphorylation of kinases and more release of Ca^2+^ from ryanodine receptors (RyRs) in endoplasmic reticulum (ER) through a calcium-induced calcium-release (CICR) thus amplifying calcium signaling. In Young synapses, HFS leads to influx of Ca^2+^ through NMDA-receptors primarily. This leads to activation of kinases that are important for LTP and learning and memory. NMDA activation also leads to activation of reactive oxygen species (ROS), which in turn increases Ca^2+^ concentration through the modulation of the activity of NMDA-receptors and RyRs. While in aged synapses compared to young LTP is altered due to (1) HFS induces Ca^2+^ influx occurs via LTCCs primarily and this shift from NMDA to LTCC causes Ca^2+^ dyshomoestasis. (2) The release of Ca^2+^ through intracellular stores like RyRs on ER also contributes to this and is facilitated by (3) ROS due to excessive oxidative stress. CPAMPAR also contributes to the calcium load in the post synaptic cell. Increased LTCC and aberrant NMDA-receptor function impair Ca^2+^ homeostasis, and is further enhanced by a decrease in activity of Ca^2+^ buffering proteins, driving an (4) altered gene expression which results in Ca^2+^dysregulation, leading to cognitive decline and neurodegeneration. Increased LTCCs and calcium-dependent potassium channels produce an (5) increase of the slow component after hyperpolarization (sAHP), which also contributes to the altered gene expression, impaired LTP or increased threshold to induce LTP (Made using Biorender).

### The role of inflammation in cognitive decline in ageing

Ageing is characterized or caused by systemic inflammation, immunosenescence (overall decline in protective immune responses) and inflammageing (chronic inflammation in the absence of overt infection). The ageing brain transitions from a state of relatively competent immune function to a state of heightened immune activation (Buchanan et al., 2008). Age-related increase in inflammation partially contribute to cognitive decline in ageing (Dilger and Johnson, 2008). Age-associated alterations in immune functions also render the ageing brain more vulnerable to diseases (Castle, 2000). This is corroborated by the association between inflammatory markers and pathological hallmarks of various diseases, like AD, PD, and mild cognitive impairment (MCI). Postmortem brains of people with late-stage AD, have revealed that beta-amyloid plaques, are frequently colocalized with several inflammatory factors, including proinflammatory cytokines and activated microglia (Eikelenboom and Van Gool, 2004; Eikelenboom et al., 2006). It remains unclear whether neuroinflammatory events precede the initiation of disease conditions or are a direct consequence of the pathological processes (Halliday et al., 2000).

Systemic inflammation in the brain leads to impairments in a variety of cognitive functions (Frank et al., 2010). Mice overexpressing the proinflammatory cytokine interleukin (IL)-1 (Moore et al., 2009) and rats subjected to the continuous ventricular administration of lipopolysaccharide (LPS) (Rosi et al., 2006), a well-known proinflammatory trigger, exhibit impairments in spatial working memory. The activation of microglia by LPS significantly enhances the expression of certain immune factors and disrupts hippocampal LTP through NMDA-receptor-mediated metaplasticity (Izumi et al., 2021). Given the connections between ageing, cognitive decline and inflammation, it’s not surprising that ageing tends to exacerbate the impact of neuroinflammation on learning and memory.

Some of the therapeutic targets of neuroinflammation include non-steroidal anti-inflammatory drugs (NSAID), which act by inhibiting the cyclooxygenase enzymes COX-1 and COX-2, are associated with a decreased risk of AD in typical ageing populations. This protective effect aligns with a prolonged preclinical period that can last for many years or even decades before the manifestation of cognitive decline (Marsland et al., 2006; Trepanier and Milgram, 2010). Estrogens, particularly agonists for estrogen receptor (ER) ERbeta, have attracted considerable attention due to their anti-inflammatory properties, which involve reducing the expression of IL-1beta and TNF-alpha. Moreover, estrogen replacement is shown to improve cognitive function in aged rhesus monkeys (Rapp et al., 2003; Hughes et al., 2009). Endocannabinoids are lipid molecules that interact with cannabinoid receptors, CB1 and CB2, and activation of CB2 receptors inhibits the expression of proinflammatory cytokines like TNF-alpha, IL-1beta, IL-6, and IL-8, increasing the expression of anti-inflammatory cytokines (Wolf et al., 2008). Interestingly, chronic activation of CB2 receptors increases excitatory synaptic transmission and reverses amyloid-induced cognitive decline (Wu et al., 2013; Kim and Li, 2015). One of the most consistently confirmed discoveries in ageing is that caloric restriction (CR), which decelerates the ageing process, diminishes inflammation in both the peripheral and central nervous systems (Colman et al., 2009). CR can reverse age-associated impairments in LTP and NMDA receptor expression (Eckles-Smith et al., 2000). Enhancing autophagy can also reduce inflammation by removing inflammasomes or inhibiting transcriptional modulators of cytokines, highlighting autophagy as a promising target for the regulation of neuroinflammation (Netea-Maier et al., 2016). Enhancing autophagy can reverse age-related cognitive functions, making it another possible target for healthy ageing interventions (Glatigny et al., 2019).

### Autophagy and brain ageing

Autophagy is a highly conserved cellular process that removes molecules and subcellular elements like misfolded or aggregated proteins and damaged organelles through a lysosome-mediated process to promote homeostasis. As individuals age, there is a reduction in autophagy, and this gradual decline in autophagy may have a causative role in the functional decline of biological systems during the ageing process. Consistently, many genes that control autophagy are linked to lifespan regulation (Lionaki et al., 2013). An age-related decline in cognitive functions are attributed to reduction in autophagy. Induction of autophagy is important for the degradation of Aβ, tau and α-syn, all of which disrupt cognitive functions during ageing (Yue et al., 2009). Promoting autophagy can enhance longevity and their repression shortens the lifespan in mice, flies and worms (Simonsen et al., 2008; Pyo et al., 2013; Nakamura and Yoshimori, 2018). Enhancing autophagy itself can have a profound influence on hippocampus dependent memory during ageing (Glatigny et al., 2019). Systemic administration of young plasma ameliorates age-related memory deficits through rehabilitating synaptic plasticity capacity in old hippocampus by restoring autophagy (Villeda et al., 2011). Conversely, memory related stimuli upregulate autophagy and that, in turn, promotes memory formation and enhances dendritic spines by modulating the adaptive response to novel stimuli in the hippocampus (Glatigny et al., 2019). Studies on flies shows that cognitive decline in ageing is associated with autophagy (Maruzs et al., 2019). Spermidine-induced autophagy reduces the aggregation of ubiquitinated proteins and thereby reduces cognitive impairment. It is also known to promote longevity (Gupta et al., 2013).

Learning induces hippocampal autophagy and drugs that inhibit or activate autophagy impair or facilitate long-term memory, respectively (Hylin et al., 2018). Acute autophagy inhibition, while not affecting basal synaptic transmission, significantly impairs hippocampal LTP and reduces paired-pulse facilitation, a short-term plasticity mechanism with a presynaptic role. Spermidine, which induces autophagy, is effective in improving hippocampal-dependent memory in elderly people with MCI (Wirth et al., 2019).

Recent studies, particularly in *C. elegans* and mammals, suggest that autophagy serves as one of the convergent downstream mechanism underlying various longevity paradigms (Pyo et al., 2013; Nakamura and Yoshimori, 2018). For example,reduction of Insulin/IGF-1 signaling, reduced mTOR signaling, calorie restriction or pharmacological activation with spermidine, reservatrol, rapamycin, or tomatidine that increases longevity and enhances autophagy (Meléndez et al., 2003; Jia and Levine, 2007; Hansen et al., 2008; Eisenberg et al., 2009; Bjedov et al., 2010; Fang et al., 2017).

While compounds known to enhance autophagy are associated with memory improvement, the mechanisms by which they influence memory remain unknown. This provides a promising avenue for studying the cellular and molecular mechanisms of autophagy-inducing drugs in alleviating memory decline in aged animal models.

### Calcium homoeostasis and ageing synapses

Calcium plays an integral role in the regulation of various processes including autophagy in the brain, structural and functional plasticity, and neurotransmitter release, activation of kinases and phosphatases, and neuronal excitability. This crucial role of calcium dictates the need for precise regulation and therefore subtle alterations could lead to major changes in cellular function. Brain ageing is largely associated with alterations in calcium homoeostasis. Alterations in various Ca^2+^ regulation mechanisms not only serve as biomarkers of ageing, but are also closely associated with cognitive decline. The age-related plasticity deficit has been linked to alterations in calcium regulation (Moore et al., 2023).

At the level of synapsis, the magnitude and direction of plasticity is determined by the level and duration of an increase in intracellular Ca^2+^. LTP is induced by a brief, larger rise in postsynaptic Ca^2+^, while LTD is induced by a modest and prolonged rise in intracellular Ca^2+^. The idea that ageing is due to elevated resting Ca^2+^ levels has been replaced by evidence for a shift in the sources of intracellular Ca^2+^. Ageing is associated with a diminished role for N-methyl-D-aspartate (NMDA)-receptors and an increased role for L-type voltage-dependent Ca^2+^ channels (LTCCs) and intracellular calcium stores (ICS). This is supported by the finding that Calcineurin (CaN) levels increases with ageing and CaN is known to dephosphorylate NMDA-receptors, reducing its function (Lieberman and Mody, 1994; Wang and Salter, 1994). Age-related increases in oxidative stress leads to increase in LTCC and ICS and a reduction in NMDA-receptors. Ca^2+^ from LTCCs and ICS during oxidative stress can in turn activate CaN, which further reduces NMDA-receptor activation. This decrease in NMDA-receptors with ageing explains the increased threshold for LTP induction (Barnes et al., 2000). The magnitude of LTP does not change with high frequency stimulation, while impairments are observed with weaker stimulus paradigms. Ageing is also characterised by increased susceptibility to LTD which is associated with forgetting of hippocampal dependent memory (Norris et al., 1996).

Although, only subtle changes are observed in the LTP induction with a shift from NMDA-receptors to ICS and LTCCs, it has a large functional significance. Synapse specificity, a basis of Hebb’s postulate as the neural substrate of learning, is impaired in ageing. High frequency stimulation of afferent fibres in aged mice induces an LTP that is not input specific and this can be reversed by blocking either LTCC or calcium-induced Ca^2+^ release (CICR) (Ris and Godaux, 2007). An increase in the afterhyperpolarization (AHP) was also observed with increased calcium levels and this, in turn, inhibits NMDA receptor activation as a feedback loop. Blockade of LTCCs also reduces AHP (Disterhoft et al., 2004), facilitating LTP induction (Norris et al., 1998). As AMPA receptor GluA2 subunit defects are a major cause of AD and neurodevelopmental disorders like autism spectrum disorders, it might be possible that calcium permeable AMPA (CPAMPA) receptors also contribute to calcium influx (Whitehead et al., 2017; Salpietro et al., 2019). Thus CPAMPAR also provides a novel outlook onto the pathophysiology of AD, considering its role in the context of amyloid-beta mediation.(Whitehead et al., 2017).

CICR from endoplasmic reticulum (ER) stores through both the Inositol trisphosphate receptor (InsP3R) and ryanodine-receptors (RyRs) occurs in aged neurons, indicating that ryanodine release might be used as a biomarker of functional ageing (Toescu, 2005; Gant et al., 2006). This Ca^2+^-induced Ca^2+^ release (CICR) by RyRs on ER is triggered by Ca^2+^ influx via LTCCs (Chavis et al., 1996; Empson and Galione, 1997; Fagni et al., 2000; Sukhareva et al., 2002; Sajikumar et al., 2009; Li et al., 2014, 2017). Early interventions using nootropic drugs that increase synaptic transmission, steroids that indirectly influence Ca^2+^-dependent processes, or hormonal therapies to decrease the oxidative damage that further alleviates age-associated calcium dysregulation are promising. Understanding the alterations in neuronal Ca^2+^ signaling and synaptic plasticity mechanisms linked to ageing is essential for the development of highly efficient therapeutics addressing brain disorders, particularly age-related cognitive impairment.

### The brain as a modulator of longevity

The concept that the CNS controls systemic ageing came from studies of longevity genes in model organisms such as *C. elegans*, *D. melanogaster*, and mice (Bansal et al., 2015; Piper and Partridge, 2018; Zullo et al., 2019). For instance, restoring Insulin signaling in neurons alone was sufficient to increase lifespan in *C. elegans* (Wolkow et al., 2000). Insulin and insulin-like growth factor (IGF) signaling (IIS) is an evolutionarily conserved pathway that plays a major role in the longevity. The regulation of IRS2 signaling in the brain shows a connection between the extension of lifespan and the regulation of metabolism. Reduced insulin signaling throughout the body or just in the brain can extend lifespan up to 18% by promoting healthy metabolism (Holzenberger et al., 2003; Taguchi et al., 2007).

Reducing IIS is a promising approach to extend lifespan as the process of brain ageing is normally associated with a decrease of cortical insulin concentration as well as impairment of insulin receptor binding ability (Frölich et al., 1998). Mice with overexpression of Klotho can inhibit IIS and extend lifespan, while the Klotho hypomorphs exhibit premature ageing perturbing insulin and IGF1 signaling, suggesting that Klotho-mediated inhibition of insulin and IGF1 signaling contributes to its anti-ageing properties (Kurosu et al., 2005). Insulin receptor null mice (Irs1−/−) had longer lifespan and displayed resistance to a range of age-sensitive biomarkers of ageing (Selman et al., 2008). In humans, deficiency in IIS have been linked to diabetes and insulin resistance (Taniguchi et al., 2006) and increased risk of cardiovascular disease (Besson et al., 2003). Preserved insulin sensitivity has been linked to longevity in human centenarians compared to middle-aged subjects, as they exhibited preserved glucose tolerance and insulin action (Fülöp et al., 1987; Paolisso et al., 1996). While insulin and IGF signaling are known for their broad impacts on metabolism and growth, genetic manipulation studies in insulin/IGF1 signaling shows that they cause insulin resistance and hyperglycemia, while extending longevity (Taguchi et al., 2007; Kappeler et al., 2008). Thus it is crucial to identify the neural circuits that mediate beneficial effects of the reduced insulin/IGF1 signaling on lifespan extension, while maintaining brain energy homeostasis and peripheral glucose metabolism.

The DAF-16/forkhead transcription factor, a downstream target of the IIS, is an important target in regulating lifespan. Concurrently, c-Jun N-terminal kinase (JNK) operates in parallel with the insulin-like signaling pathway to modulate lifespan, with both pathways converging onto DAF-16. In flies, activation of c-Jun N-terminal kinase (JNK) signaling in neurons increased lifespan, in part through FOXO-mediated transcription (Lee et al., 2009).

In humans, a study has shown that longevity is associated with the downregulation of genes linked to neural excitation and synaptic function. The transcription factor NRSF/REST is upregulated in brains who had an extended lifespan. It extends lifespan by repressing the excitation-related genes (Zullo et al., 2019). This again represent a mechanism of increasing longevity by targeting a neural circuit. NRSF/REST plays an important role in the preservation of memory and activity-dependent synaptic plasticity during ageing (Yang et al., 2023).

AMP-activated protein kinase (AMPK) activation is another signaling pathway that was shown to prolong lifespan and delay ageing (Salminen and Kaarniranta, 2012; Grahame Hardie, 2016). The AMPK controls the regulation of metabolism, stress resistance, cellular homeostasis, cell survival and growth, cell death and autophagy which are some of the important regulators of ageing and lifespan (Salminen and Kaarniranta, 2012). A decrease in AMPK activation and its responsiveness with age, explains the altered metabolic regulation, resulting in reduced autophagy and an increase in oxidative stress (Salminen and Kaarniranta, 2012). Although it is known to extend lifespan, whether neuronal AMPK activity in the brain alone is sufficient to prolong lifespan is not fully understood. A polyphenol resveratrol that activated AMPK increased both median and maximum lifespan in mammalian neurons and delayed age-dependent decay in cognitive performance in fish *Nothobranchius furzeri* (Valenzano et al., 2006). AMPK inhibits mTOR, and downregulation of mTOR has been shown to promote lifespan in several organisms and thus regulation of mTOR by AMPK provides another likely mechanism by which AMPK could influence lifespan (Jia et al., 2004; Kapahi et al., 2010; Saxton and Sabatini, 2017). Clinical trials with AMPK activators like resveratrol, metformin and exercise, are investigating their effects on human ageing-related characteristics, tissue homeostasis, and metabolic dysfunctions (Chen et al., 2015; Duca et al., 2015; Fentz et al., 2015).

Sirtuins, are a class of Nicotinamide adenine dinucleotide (NAD^+^)-dependent protein deacetylases that are known to regulate various cellular processes including DNA repair, inflammation, and metabolism. In the brain, sirtuins are implicated in maintaining neuronal health and promoting longevity by enhancing cellular stress resistance and improving mitochondrial function (Ng et al., 2015). Overexpression of brain-specific Sirt1 in mice delays ageing and increases lifespan and is particularly interesting as SIRT1 may influences ageing through its activities in the brain (Satoh et al., 2013). The significant lifespan extension was seen in both males and females and are mediated by increased neural activity specifically in the dorsomedial and lateral hypothalamic nuclei through increased orexin type 2 receptor expression. Sirtuins also influences the longevity via modulating IIS (Satoh et al., 2013; Zhao et al., 2020). Calorie restriction also increases the level of most sirtuins, except SIRT4 (Wątroba and Szukiewicz, 2016). Sirtuins interact with all the major conserved longevity pathways, like AMPK, Insulin signaling, and targets like PKA, mTOR, FOXO etc. Thus although, Sirtuins, as potential targets for interventions promoting healthy ageing and longevity remains an exciting area of exploration in the field of longevity science more studies need to be done to prove its effects in humans.

Nuclear factor erythroid-derived 2-like 2 (NRF2; encoded by the *Nfe2l2* gene), a member of the cap‘n’collar (CNC) protein family is yet another pathway that is involved in longevity extension. NRF2 signaling decreases with age and silencing NRF2 leads to premature senescence (Fulop et al., 2018). Transient activation of NRF2 in endothelial progenitor cells from aged mice protected these cells against oxidative stress and downregulated the NLR family pyrin domain containing 3 (NLRP3) inflammasome (Wang et al., 2019). But a persistent activation induced cellular senescence potentially showing why NRF2 is so tightly regulated at multiple levels of gene expression (Hiebert et al., 2018). While Nrf2 is present in neurons, astrocytes, and microglial cells, its activity is significantly higher in astrocytes and microglia compared to neurons. NRF2 increases glutathione synthesis in the brain that protects neurons against oxidative damage and provides beneficial effects (Cuadrado, 2022). Cognitive function in mice models of AD has been demonstrated to improve with the administration of NRF2-activating chemicals such as CDDO-methyl-amide and dimethyl fumarate (Uruno et al., 2020). This could hold future promise as control of neuroinflammation, in addition to suppression of oxidative stress, appears to be necessary to delay ageing and prevent neurodegenerative diseases (Table 1).

**Table 1:** Summary table showing comparison of young versus ageing brain.

	Young brain	Ageing brain	
Cognition	Cognitive development (Murman, 2015)	Decline in cognitive functions (Rosenzweig and Barnes, 2003; Murman, 2015)	
Neuroinflammation	Systemic inflammation (Sankowski et al., 2015)	Chronic inflammation (Shastri et al., 2013; Andronie-Cioara et al., 2023; Gamage et al., 2023)	
Neurogenesis	Intact neurogenesis (Wu et al., 2023)	Slow decline in neurogenesis (Wu et al., 2023)	
LTP	Intact LTP (Lu et al., 2024)	Reduction in the magnitude of LTP (Increased threshold for inducing LTP) (Wong et al., 2021; Wang et al., 2023)	
Synaptic tagging	Intact synaptic tagging/ capture (Shivarama Shetty and Sajikumar, 2017)	Impaired synaptic tagging/capture (Sharma et al., 2015; Shetty et al., 2017; Shivarama Shetty and Sajikumar, 2017)	
LTD	Decreased sensitivity to LTD/NMDA -receptor dependent LTD (Rajani et al., 2021)	Increased sensitivity to LTD / mGLuR (metabotropic glutamate receptor) dependent LTD (Norris et al., 1996; Temido-Ferreira et al., 2020)	
NMDA	NMDA-receptor dependent LTP/LTD (Thibault et al., 1998)	NMDA-receptor independent-LTP (Thibault et al., 1998)	
LTCC	LTP independent of LTCC (L-type calcium channels) (Moyer et al., 1992)	LTP dependent on LTCC (Moyer et al., 1992; Thibault and Landfield, 1996)	
Calcium	Balanced calcium homoeostasis (Huidobro et al., 1993)	Increased calcium and after hyperpolarization (AHP) (Foster, 2007; Oh et al., 2013)	
Autophagy	Balanced autophagy (Aman et al., 2021)	Compromised autophagy (Aman et al., 2021; Tabibzadeh, 2023)	

In recent years, a compelling area of research has emerged, focusing on the influence of FOXO transcription factors on longevity. FOXO (Forkhead Box O) transcription factors are evolutionarily conserved from *C.elegans* to mammals and play critical roles in diverse biological processes, in particular ageing and longevity. Foxo factors act as transcriptional effectors of the IIS, regulate genes responsible for autophagy and the ubiquitin-proteasome system and maintain intracellular homoestasis by increasing the antioxidant capacity of cells (Kenyon et al., 1993; Kops et al., 2002; Webb and Brunet, 2014). DAF/FOXO acts by integrating signals from various pathways to regulate the processes of ageing and longevity. Insulin-like molecules bind to the DAF-2 receptor, initiating the activation of the PI3P pathway, which in turn, inhibits the translocation of DAF-16/FOXO into the nucleus. JNK and AMPK pathway activates FOXO, while TOR inhibits it. Thus, DAF-16/FOXO integrates signals from these multiple pathways, shuttling from the cytoplasm to the nucleus to modulate the processes of ageing and longevity. Therefore, unraveling the functional mechanisms of DAF-16/FOXO is crucial for gaining insights into the intricate dynamics of ageing and longevity.

Brain-Derived Neurotrophic Factor (BDNF) that plays a pivotal role in facilitating plastic changes associated with learning and memory might be another important player in longevity. Lifestyle changes, particularly calorie restriction and exercise that are known to extend lifespan improves cognitive functions by increasing BDNF levels (Kishi et al., 2015; Sleiman et al., 2016). In healthy old adults, 35-min sessions of physical exercise, cognitive training, or mindfulness practice, increased BDNF levels (Håkansson et al., 2017). Modulation of autophagy by BDNF signaling pathway underlies synaptic plasticity (Nikoletopoulou et al., 2017). Moreover, metformin alleviates age-induced neurocognitive deficits via the activation of the AMPK/BDNF/PI3K pathway (Ameen et al., 2022). Studies showing a novel role for CREB-regulated transcriptional coactivators and CREB in determining lifespan downstream of AMPK and calcineurin also strengthens our view on BDNF as another possible link as CREB signaling regulates expression of genes that promotes BDNF (Mair et al., 2011). Alterations in BDNF expression have implications for both normal and pathological ageing, particularly in the hippocampus and parahippocampal areas (Miranda et al., 2019). The modulation of BDNF levels through dietary or pharmacological interventions that affect longevity highlights the importance of BDNF as a molecular target in understanding and potentially promoting longevity.

### The dynamic brain and neuroplasticity and the role of lifestyle in shaping synaptic plasticity

Interventions in ageing by lifestyle changes or therapeutic strategies that promote hippocampal rejuvenation to restore synaptic plasticity and cognitive functions hold promise in preventing age associated memory decline. For example, exercise can preserve hippocampal function with age. In rodents, better acquisition and retention of memory in a Morris water maze, inhibitory avoidance, contextual fear, and object recognition were observed after long-term voluntary running in aged animals (Kumar et al., 2012; Speisman et al., 2013; Gibbons et al., 2014). In humans, cardiovascular exercise is associated with more accurate spatial short-term memory and less hippocampal atrophy (Voss et al., 2010; Erickson et al., 2011; Duzel et al., 2016). Exercise was shown to increases LTP and lower the threshold for LTP induction (Loprinzi et al., 2019). The effect of exercise training on LTP and NMDA receptor channels in rats with cerebral infarction shows that LTP and synaptic efficacy in the hippocampal CA3 area after exercise training in the rehabilitation group was significantly higher compared to controls without any exercise training (Yu et al., 2013). These cognitive improvements are mediated by changes in plasticity, neurogenesis and inflammation. In rodents, running also reverses age associated LTP impairments in dentate gyrus by increasing neurogenesis (Wu et al., 2015; Duzel et al., 2016). In elderly humans, running enhances blood flow in the hippocampus (Burdette et al., 2010). Exercise induces the release of cathepsin B (CTSB) from muscles, which increases BDNF expression, neurogenesis, and memory performance (Moon et al., 2016).

Parabiosis studies have shown that exposure to young blood rescued age-related decline in LTP, increased dendritic spine number, and promoted neuronal activity in the aged hippocampus, as evidenced by increased c-fos expression and CREB phosphorylation (Villeda et al., 2014). Administration of young plasma to aged animals also reversed hippocampus dependent memory impairments (Villeda et al., 2011, 2014). This shows that systemic factors can also modulate the functioning of the ageing brain. This area is very promising as it provides a framework for clinical interventions to prevent neurodegenerative diseases in addition to normal age-related disorders.

Numerous studies have documented neuroplastic alterations within the brains of healthy individuals resulting from routine processes, such as learning (Pascual-Leone et al., 2005; Warraich and Kleim, 2010; May, 2011). In a longitudinal study involving London taxi drivers, the acquisition of an internal spatial representation of London was associated with a selective increase in gray matter in the posterior hippocampus (Woollett and Maguire, 2011). This shows structural changes in the brain can be induced by biologically relevant behaviors that involved cognitive functions, such as spatial memory. Similarly, mental rehearsal of a task alone could lead to neuroplastic changes in the brain (Warraich and Kleim, 2010). Individuals who are blind or deaf frequently exhibit enhanced abilities in their remaining senses. This indicates that the absence of a sensory modality uniquely exemplifies the brain’s capacity to effectively reorganize the allocation of its resources in response to demands and to compensate for deficits (Merabet and Pascual-Leone, 2010; Ricciardi and Pietrini, 2011). Following sensory deprivation, such as visual or auditory deprivation, brain areas that are normally associated with the lost senses are taken over by spared sensory modalities. This underlies the adaptive and compensatory mechanisms that neuroplasticity entails to cope with sensory loss (Merabet and Pascual-Leone, 2010).

Nutrition has an enormous effect in shaping neuroplasticity and preventing cognitive decline. A study on Long Evans rats showed that compared to animals who were on a balanced diet, the rats who were on a diet high in saturated fat and cholesterol had more working memory errors and was even more significant when they were intellectually challenged with higher memory tests (Mattson, 2010). High-fat diets impairs hippocampus dependent memory by increasing glutamate uptake, decreasing synaptic efficacy and inhibits LTP and NMDA-receptor dependent LTD (Contreras et al., 2017). Intermittent fasting (IF) alters metaplastic properties of CA1 hippocampal neurons and facilitate synaptic tagging/capture due to increased neurogenesis and upregulation of BDNF and Prkcz, in IF (Dasgupta et al., 2018). A periodic diet similar to IF promotes cognitive performance and healthspan in mice (Brandhorst et al., 2015).

Change in sleep patterns or sleep deprivation (SD) is another hallmark of ageing (Gooneratne and Vitiello, 2014). SD is known to impair synaptic plasticity and memory (Campbell et al., 2002; Goel et al., 2009). Rapid eye movement (REM) SD has also been shown to interfere with LTP and learning and memory (Kim et al., 2005). Ted Abel’s lab showed that a brief SD impairs hippocampal LTP by interfering with cAMP signaling through enhanced PDE4 activity. Thus, interfering with cAMP signaling may provide promising approaches to counteract the cognitive effects of SD (Vecsey et al., 2009). Studies also showed that SD impairs synaptic tagging and capture of proteins (Wong et al., 2019). The sleep patterns of people who live longer show that they go to bed early, wake up early, and take a nap in the afternoon, indicating that the quality of sleep has a significant influence on longevity (Mazzotti et al., 2014).

Apart from these, quality of life also has a profound effect on longevity. Although mild stress is known to increase both memory and longevity (Minois, 2000; Ahmed et al., 2006; Corbett et al., 2017) chronic stress can accelerate ageing (Yegorov et al., 2020). Ageing alters homoeostasis by altering endocrine, autonomic, and immune systems. Several studies have shown that a rise in corticosteroid level causes reduction in hippocampal LTP (Joëls and Krugers, 2007).

Brain ageing is a surprisingly dynamic process, implying that interventions or strategies could be developed for preserving cognitive function and potentially restoring cognitive abilities that may have declined over time. Re-establishing these connections and reinstating their functions by changing lifestyle holds particular significance within the field of cognitive ageing and can be employed to enhance functionality in the context of healthy ageing.

### Comparative ageing of human, rodents and non-human primates as models for ageing research

The average lifespan of a particular species is defined as the maximum number of years a member of that species has been known or expected to survive. For example, the average human lives about 80 years, while the average lifespan of a laboratory mouse is about 2 years (Dutta and Sengupta, 2016). Comparing biological age across species involves assessing the progression of biological changes related to aging, rather than simply calculating the proportion of the average lifespan that has been lived.

Extensive evidence supports the laboratory mouse as the primary model for studying human aging. However, little is known regarding their behavioral and functional changes that occur throughout their lifespan and how these correspond to the aging phenotype observed in humans. As mice have a significantly shorter lifespan compared to humans it is crucial to establish the precise age correlation between mice and humans at different stages of their lifespan to create experimental mouse models that resemble human age groups. For example the maturational rate of mice does not linearly correlate with that of humans. Mature adult mice, aged 3 to 6 months, are roughly equivalent to humans aged 20 to 30 years. Mice should be at least 10 months old to be considered middle-aged, corresponding to human ages of 38 to 47 years. Mice aged 18 to 24 months correlate with humans aged 56 to 69 years, an age range defined as “old or aged” due to the presence of senescent changes. Mice older than 24 months can be considered “very old,” a point at which survivorship drops off markedly.

Rodents are valuable models for aging research due to their similarities to humans in the physiology and cell biology of aging. However, significant differences between rodents and humans necessitate the use of models that are closer to humans. Compared to rodents, non-human primates (NHPs) are more closely related to humans and better replicate the aging and disease mechanisms in humans (Colman, 2018). They are also a good model for ageing translational research. NHPs like monkeys aged 20 years and over correspond to humans aged 60 and over, while monkeys aged 30 years and over correspond to humans in their 90s or older. Marmosets have a relatively shorter lifespan compared to monkeys with an average lifespan of 5 to 7 years and maximum of 16.5 years (Abbott et al., 2003). Yet another important factor in while selecting an appropriate model would be to look at the similarities of the disease pathogenesis as well as whether the species has similar biomarkers as the human disease (Mattison and Vaughan, 2017). As such the use of animal models in aging research is contributing to human health by advancing our understanding of the molecular, cellular, biochemical, and behavioral mechanisms of aging.

## Author contributions

SN: Writing – original draft, Writing – review & editing. BK: Writing – original draft, Writing – review & editing.
